# Admission EASIX Score Predicts Coronary No-Reflow and In-Hospital Mortality in STEMI Patients Undergoing Primary PCI

**DOI:** 10.3390/jcm15031063

**Published:** 2026-01-29

**Authors:** Yusuf Bozkurt Şahin, Veysel Ozan Tanık, Sinan Boz, Murat Akdoğan, Çağatay Tunca, Özden Seçkin, Alperen Taş, Bülent Özlek

**Affiliations:** 1 Department of Cardiology, Ankara Etlik City Hospital, 06170 Ankara, Türkiye; drozantanik@gmail.com (V.O.T.); drsinanboz@gmail.com (S.B.); drmuratakdogann@gmail.com (M.A.); md.tunca@gmail.com (Ç.T.); 2 Department of Cardiology, Faculty of Medicine, Gazi University, 06500 Ankara, Türkiye; ozden-seckin@hotmail.com; 3 Department of Cardiology, Kırşehir Training and Research Hospital, 40200 Kırşehir, Türkiye; alperentas555@hotmail.com; 4 Department of Cardiology, Faculty of Medicine, Muğla Sıtkı Koçman University, 48000 Muğla, Türkiye; bulent_ozlek@hotmail.com

**Keywords:** endothelial activation and stress index, in-hospital mortality, no-reflow phenomenon, primary PCI, STEMI

## Abstract

**Background:** Early risk stratification in ST-elevation myocardial infarction (STEMI) remains critical, particularly for anticipating adverse outcomes such as the coronary no-reflow phenomenon (NRP) and early mortality. The Endothelial Activation and Stress Index (EASIX), calculated from routine laboratory parameters, has emerged as a potential biomarker reflecting systemic endothelial dysfunction. This study evaluated the prognostic value of admission EASIX for the NRP and in-hospital mortality in STEMI patients undergoing primary percutaneous coronary intervention (pPCI). **Methods:** In this retrospective single-center cohort, 1931 STEMI patients treated with pPCI between January 2023 and January 2025 were included. EASIX was calculated at admission. NRP was defined as post-PCI TIMI flow ≤ 2 or TIMI 3 flow with impaired myocardial blush (TMPG ≤ 1). Multivariable logistic regression, reclassification analyses (NRI/IDI), ROC analysis, and calibration methods were used to assess predictive performance. Sensitivity and interaction analyses were conducted. **Results:** NRP occurred in 14.1%, and in-hospital mortality was 2.5%. EASIX was independently associated with both outcomes (NRP: adjusted OR 1.485, 95% CI 1.286–1.715; mortality: adjusted OR range 1.371–2.096 across models; all *p* < 0.001). EASIX significantly improved risk reclassification for both NRP and in-hospital mortality (NRI > 0.20). ROC-AUCs were 0.706 for NRP and 0.810 for mortality. Restricted cubic spline and LOWESS analyses revealed nonlinear risk escalation. Calibration plots and Brier scores confirmed model reliability. Associations persisted across ischemic time and renal function strata. **Conclusions:** Admission EASIX is independently associated with NRP and in-hospital mortality in STEMI. Easily accessible and integrative, EASIX may enhance early risk stratification. External validation is warranted before clinical implementation.

## 1. Introduction

Cardiovascular diseases remain the leading cause of mortality worldwide [1]. Among them, ST-segment elevation myocardial infarction (STEMI) is a life-threatening condition in which prompt diagnosis and rapid restoration of coronary blood flow are essential to minimize myocardial injury and preserve left ventricular function [2]. Primary percutaneous coronary intervention (pPCI) is the preferred reperfusion strategy for STEMI and has markedly improved survival rates [2]. Nevertheless, successful restoration of epicardial coronary patency does not always translate into effective myocardial tissue perfusion, highlighting a persistent gap between angiographic success and true microvascular reperfusion [3].

Despite substantial advances in interventional techniques and adjunctive pharmacotherapy, the no-reflow phenomenon (NRP) remains a frequent and clinically significant complication after pPCI [4]. NRP is defined as impaired myocardial perfusion despite successful reopening of the infarct-related artery and is primarily attributed to microvascular dysfunction rather than residual epicardial obstruction [5]. Depending on the diagnostic modality and criteria used, the incidence of NRP has been reported to range from 2% to 44% [6]. Importantly, NRP has been consistently associated with adverse clinical outcomes, including larger infarct size, impaired left ventricular remodeling, progressive heart failure (HF), and increased short- and long-term mortality [7].

Endothelial dysfunction plays a pivotal role in microvascular injury and has emerged as a key determinant of adverse cardiovascular outcomes [8]. Characterized by oxidative stress, inflammatory cytokine release, and prothrombotic activation, endothelial dysfunction closely mirrors the biological mechanisms implicated in NRP [9]. In this context, the Endothelial Activation and Stress Index (EASIX) has been proposed as a simple, reproducible, and cost-effective biomarker of systemic endothelial stress. Derived from routinely available laboratory parameters—lactate dehydrogenase (LDH), creatinine, and platelet count—EASIX integrates markers of cellular injury, renal impairment, and platelet consumption into a single score that quantifies endothelial distress [10].

Given that NRP is a clinical consequence of profound endothelial injury and microvascular dysfunction, evaluating EASIX in STEMI may provide a pathophysiologically relevant and clinically practical tool for early risk stratification. Beyond its potential role in predicting NRP, heightened systemic endothelial stress may also contribute to early adverse outcomes, including in-hospital mortality, after STEMI. Therefore, the present study aimed to investigate the predictive value of the EASIX score for NRP and in-hospital mortality among patients with STEMI undergoing pPCI.

## 2. Methods

### 2.1. Study Design and Patient Population

This retrospective, single-center observational study enrolled patients presenting with STEMI who underwent PCI between January 2023 and January 2025 at Ankara Etlik City Hospital, a high-volume tertiary cardiovascular referral center with a 24 h catheterization laboratory. A total of 1931 patients aged ≥18 years were included. STEMI was diagnosed according to the European Society of Cardiology (ESC) guidelines [11]. All eligible patients underwent emergent coronary angiography (CAG) and pPCI with iodinated contrast administration.

### 2.2. Exclusion Criteria

Patients were excluded if they had received fibrinolytic therapy before hospital admission, had missing data (*n* = 21), or had clinical conditions that could confound endothelial or inflammatory status. These included known chronic inflammatory or autoimmune diseases (e.g., rheumatoid arthritis, systemic lupus erythematosus, systemic vasculitis) (*n* = 7), active infection or sepsis (*n* = 5), severe hepatic dysfunction or active hepatitis (*n* = 3), and known hematologic malignancies (*n* = 2).

### 2.3. Clinical Management and Follow-Up

All patients were monitored continuously from admission until hospital discharge or in-hospital death. Clinical management followed contemporary ESC recommendations for STEMI [11]. Emergency CAG and PCI were performed with a median first medical contact–to-device time of 120 min. Guideline-directed medical therapy—including dual antiplatelet therapy, statins, β-blockers, and renin–angiotensin system inhibitors—was initiated in all patients unless contraindicated.

### 2.4. Data Collection and Variable Definitions

Clinical characteristics, laboratory findings, and angiographic data were retrieved retrospectively from the institutional electronic medical record system and digital angiography archive. Collected variables included baseline demographics, cardiovascular risk factors, admission laboratory values, echocardiographic findings, and procedural characteristics. Diabetes mellitus (DM) and hypertension were defined according to current international guidelines. Left ventricular ejection fraction (LVEF) was assessed using transthoracic echocardiography and calculated by the modified biplane Simpson method. Renal function was evaluated using the estimated glomerular filtration rate (eGFR), calculated with the Chronic Kidney Disease Epidemiology Collaboration equation. Chronic renal failure (CRF) was defined as an eGFR ≤ 60 mL/min/1.73 m^2^ [12]. In accordance with our institutional STEMI protocol, all baseline demographic characteristics, comorbid conditions, laboratory parameters—including blood samples and LVEF—were systematically obtained in the emergency department prior to patient transfer to the catheterization laboratory. Transthoracic echocardiographic assessment of LVEF was routinely performed at the bedside immediately following STEMI diagnosis, before pPCI was initiated. As such, no post-procedural data were used for baseline characterization or outcome prediction analyses.

### 2.5. Angiographic Assessment and Definition of No-Reflow

Post-procedural CAG findings were independently reviewed by two experienced interventional cardiologists, who were blinded to patient outcomes and study hypotheses, and post-procedural coronary flow was evaluated using the Thrombolysis in Myocardial Infarction (TIMI) flow and TIMI Myocardial Perfusion Grading (TMPG) system [13]:

- TIMI 0: No antegrade flow beyond the occlusion;
- TIMI 1: Minimal contrast penetration without distal vessel opacification;
- TIMI 2: Complete distal vessel opacification with delayed contrast clearance;
- TIMI 3: Normal antegrade flow with rapid contrast washout.
- TMPG 0: No myocardial blush or contrast density within the infarct-related myocardial territory, indicating absence of myocardial perfusion.
- TMPG 1: Minimal myocardial blush with markedly delayed entry or persistent staining of contrast, reflecting severely impaired microvascular perfusion.
- TMPG 2: Moderate myocardial blush that is evident but clears slowly compared with normal myocardium, indicating incomplete or delayed microvascular perfusion.
- TMPG 3: Normal myocardial blush with rapid appearance and prompt clearance of contrast, comparable to that in non-infarct-related myocardial territories, indicating normal microvascular perfusion.

The NRP was defined as impaired myocardial perfusion in the infarct-related artery following technically successful PCI, indicated by either a final TIMI flow grade ≤ 2 or TIMI 3 flow with impaired myocardial perfusion (TMPG ≤ 1) [13].

### 2.6. EASIX Score Calculation

The EASIX was calculated using laboratory parameters obtained from blood samples collected at hospital admission, prior to PCI. The score was derived according to the following formula [10]:

EASIX = (LDH [U/L] × Creatinine [mg/dL])/Platelet count (10^9^/L)

Serum LDH, creatinine, and platelet counts were measured using standardized automated laboratory methods.

### 2.7. Study Outcomes

The primary outcome of the study was the occurrence of the NRP following pPCI. The secondary outcome was in-hospital all-cause mortality, defined as death from any cause occurring during the index hospitalization.

### 2.8. Ethical Considerations

The study was approved by the Ethics Committee of Ankara Etlik City Hospital (approval number: AESH-BADEK2-2025-499). All procedures adhered to the ethical standards of the Declaration of Helsinki and its subsequent revisions. Due to the retrospective and non-interventional design of the study, which involved analysis of de-identified clinical data without direct patient interaction, the requirement for written informed consent was formally waived. Patient privacy was rigorously protected, and all data were anonymized before analysis.

### 2.9. Statistical Analysis

All statistical analyses were performed using IBM SPSS Statistics software version 26.0 (IBM Corp., Armonk, NY, USA). Figures and advanced statistical visualizations (e.g., ROC curves, spline models, calibration plots) were generated using Python (version 3.11) with relevant scientific and plotting libraries (e.g., pandas, statsmodels, matplotlib, seaborn). The normality of continuous variables was assessed using the Kolmogorov–Smirnov test. Continuous variables with a normal distribution are expressed as mean ± standard deviation, whereas non-normally distributed variables are presented as median values with interquartile ranges (IQRs). Categorical variables are reported as absolute numbers and percentages. For comparisons between patients with and without the NRP, continuous variables were analyzed using the independent-samples Student’s *t*-test or the Mann–Whitney *U* test, depending on data distribution. Categorical variables were compared using the chi-square test or Fisher’s exact test when expected cell counts were less than five. To explore the association between the EASIX score and adverse outcomes, patients were stratified into three groups according to EASIX percentiles: <25th percentile, 25th–75th percentile, and >75th percentile. Continuous variables across EASIX tertiles were compared using one-way analysis of variance for normally distributed data or the Kruskal–Wallis test for non-normally distributed data, with appropriate post hoc pairwise comparisons. Categorical variables across tertiles were evaluated using the chi-square test. Univariable logistic regression analyses were initially conducted to identify variables associated with NRP. Variables demonstrating an association with the NRP at *p* < 0.10 in univariable logistic regression analyses were considered candidates for multivariable modeling. To avoid overadjustment and multicollinearity, clinical judgment and biological plausibility were applied in conjunction with statistical diagnostics. Because the EASIX is a composite score derived from LDH, serum creatinine, and platelet count, these components were not included simultaneously in the multivariable analysis. Similarly, closely related renal function parameters—including CRF and hemodialysis—were excluded in favor of eGFR as a continuous and integrative measure of renal function. In the same manner, HF was not included in the multivariable model because of its strong clinical and pathophysiological association with LVEF, which was retained to better reflect systolic function. To ensure model stability and prevent overfitting, the number of variables entered into the multivariable model was limited according to the recommended events-per-variable criterion, maintaining at least 10 outcome events per included covariate. Multicollinearity among the selected covariates was formally evaluated using variance inflation factors (VIFs). All variables in the final multivariable model had VIF values ranging from approximately 1.0 to 1.9, well below the predefined threshold for significant multicollinearity (VIF > 3.0). Variables excluded from the final model exhibited substantially higher collinearity with retained predictors or represented overlapping clinical constructs. The final multivariable logistic regression model, therefore, included age, symptom-to-balloon time, LVEF, eGFR, blood glucose, admission troponin level, white blood cell (WBC) count, and the EASIX score. For the analysis of in-hospital mortality, univariable logistic regression was initially performed to identify clinical, laboratory, and procedural variables associated with the outcome. Variables showing a univariable association with in-hospital mortality at *p* < 0.10 were considered potentially relevant predictors. However, given the relatively limited number of in-hospital mortality events, a single fully adjusted multivariable model was intentionally avoided to reduce the risk of overfitting and unstable estimates. Instead, multivariable analyses were performed using multiple domain-specific model configurations, each incorporating the EASIX and a limited number of additional covariates selected for clinical relevance, biological plausibility, and prior univariable associations. This modeling strategy allowed assessment of the robustness and independence of EASIX across different clinical contexts, including hemodynamic status, renal and metabolic function, inflammatory burden, myocardial injury, procedural characteristics, and comorbidity profiles. In accordance with the recommended events-per-variable principle, the number of predictors in each multivariable model was restricted to maintain an adequate ratio of outcome events per covariate. Variables exhibiting substantial collinearity or overlapping pathophysiological constructs—such as components of the EASIX score, closely related renal function measures, or highly interdependent clinical conditions—were not entered simultaneously into the same model. Multicollinearity was formally evaluated using VIFs, and all variables retained in the final models had low VIFs, indicating the absence of clinically meaningful collinearity. Adjusted odds ratios (ORs) with corresponding 95% confidence intervals (CIs) were reported for the EASIX score across all multivariable models to determine its independent association with in-hospital mortality. Results are reported as odds ratios (ORs) with 95% confidence intervals (CIs). The incremental prognostic value of the EASIX score was further assessed using reclassification analyses. Net reclassification improvement (NRI) and integrated discrimination improvement (IDI) were calculated by comparing baseline multivariable logistic regression models with models that included the EASIX score. Continuous (category-free) NRI was used, given the binary nature of the outcomes. These analyses were performed to evaluate improvements in risk reclassification and model discrimination after adding EASIX. Model calibration was evaluated using calibration plots and quantified by the Brier score, with lower values indicating better overall predictive performance. The discriminatory performance of the EASIX score for predicting NRP and in-hospital mortality was assessed using receiver operating characteristic (ROC) curve analysis. Optimal cut-off values were determined using Youden’s index to maximize sensitivity and specificity. Potential non-linear associations between the EASIX score and study outcomes were evaluated using restricted cubic spline analysis (RCS) and smoothed curve methods, as appropriate. For the NRP, RCS functions were applied within logistic regression models, and non-linearity was formally assessed using likelihood ratio tests comparing spline-based and linear models. For in-hospital mortality, given the limited number of events, non-linearity was explored using locally weighted smoothing (LOWESS) curves with 95% confidence bands derived from bootstrap resampling. Internal validation of the predictive models was performed using bootstrap resampling (1000 iterations) to assess model stability and robustness. Bootstrap-based procedures were applied to evaluate smoothed curve analyses and calibration metrics, allowing estimation of confidence intervals and the internal consistency of model performance. All statistical tests were two-tailed, and a *p*-value < 0.05 was considered statistically significant.

## 3. Results

### 3.1. Baseline Characteristics of the Study Population

A total of 1931 patients with STEMI undergoing pPCI were included in the study. The median age of the overall population was 58 years, and the majority of patients were male (82.9%). The median hospital stay was 3 days (IQR, 3–4 days). The NRP was developed in 272 patients, corresponding to 14.1% of the study population.

Baseline demographic, clinical, procedural, and laboratory characteristics stratified by NRP status are summarized in Table 1. Patients who developed NRP were significantly older compared with those without NRP (59 vs. 56 years, *p* = 0.001), whereas sex distribution and smoking status were similar between the two groups.

Among comorbid conditions, the prevalence rates of cardiac arrest on admission (11 vs. 4.6%, *p* < 0.001), HF (8.8 vs. 3.7%, *p* < 0.001), CRF (3.7 vs. 1.3%, *p* = 0.005), and the need for hemodialysis (3.3 vs. 0.7%, *p* < 0.001) were significantly higher in patients with NRP. In contrast, the rates of DM, hypertension, chronic obstructive pulmonary disease (COPD), and prior PCI did not differ significantly between groups.

Procedural characteristics, including culprit vessel distribution, stent length, stent diameter, and the use of tirofiban, were comparable between patients with and without NRP. However, symptom-to-balloon time was significantly longer in patients who developed NRP (median 210 vs. 180 min, *p* = 0.002).

Regarding laboratory findings, patients with NRP exhibited significantly lower LVEF (45 vs. 50%, *p* = 0.001) and worse renal function, reflected by higher serum creatinine levels and lower eGFR. Admission glucose levels, troponin, LDH, and WBC count, whereas platelet counts were significantly lower (*p* < 0.05 for all). In addition, EASIX scores were also significantly higher [1.21 (0.87–1.59) vs. 0.80 (0.59–1.12), *p* < 0.001] in the NRP group. Other laboratory parameters—including serum sodium, albumin, lipid profile, inflammatory markers, hemoglobin, and differential leukocyte counts—were similar between the two groups.

Importantly, in-hospital mortality was markedly higher among patients who developed NRP compared with those without NRP (8.5 vs. 1.5%, *p* < 0.001).

### 3.2. Association Between EASIX Score and NRP

Table 2 summarizes the results of the univariable and multivariable logistic regression analyses performed to identify predictors of the NRP. In univariable analyses, several demographic, clinical, procedural, and laboratory variables were associated with NRP; however, only selected variables were retained in the multivariable model, in accordance with predefined criteria for collinearity and biological overlap.

In the multivariable logistic regression analysis, symptom-to-balloon time remained independently associated with the development of NRP (OR 1.002 per minute, 95% CI 1.001–1.003; *p* < 0.001). In addition, admission troponin level independently predicted NRP (OR 1.012, 95% CI 1.004–1.019; *p* = 0.002), as did WBC count (OR 1.069, 95% CI 1.033–1.106; *p* < 0.001). Importantly, the EASIX score emerged as a strong independent predictor of the NRP after multivariable adjustment (OR 1.485, 95% CI 1.286–1.715; *p* < 0.001). In contrast, variables that were significant in univariable analyses—such as age, cardiac arrest on admission, LVEF, eGFR, and blood glucose level—did not retain independent significance in the multivariable model.

The incremental prognostic value of the EASIX score for predicting NRP was further evaluated using reclassification analyses. When EASIX was added to the multivariable logistic regression model that included age, symptom-to-balloon time, eGFR, blood glucose, admission troponin level, and WBC count, a positive NRI of 0.489 was observed, indicating improved risk reclassification for patients with and without NRP. In addition, the inclusion of EASIX resulted in an IDI of 0.009, reflecting a modest but favorable increase in the model’s discriminative ability.

**Figure 1:**
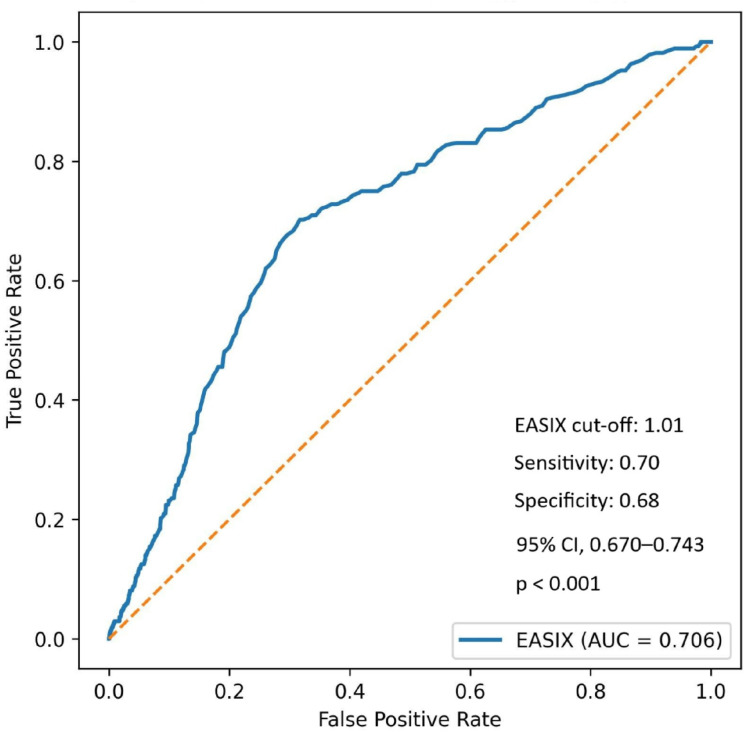
Receiver Operating Characteristic curve for the EASIX score predicting the no-reflow phenomenon (AUC = 0.706). Abbreviations: AUC, area under the curve; CI, confidence interval; EASIX, endothelial activation and stress index. The orange dashed line represents the reference line corresponding to an AUC of 0.5, indicating random classification performance.

Using Youden’s index, an optimal EASIX cut-off value of 1.01 yielded a sensitivity of 70.2% and a specificity of 68.3% for predicting the NRP. The area under the curve (AUC) was 0.706 (95% CI, 0.670–0.743; *p* < 0.001), with positive and negative predictive values of 26.6% and 93.3%, respectively (Figure 1).

**Figure 2:**
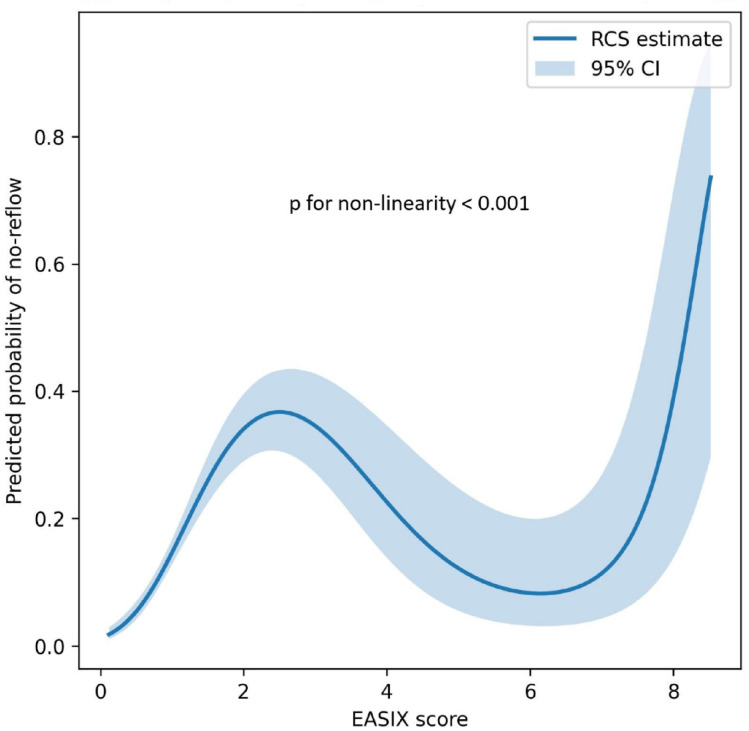
Restricted Cubic Spline (RCS) analysis of the association between the EASIX score and the probability of the no-reflow phenomenon. Abbreviations: CI, confidence interval; EASIX, endothelial activation and stress index.

In an RCS analysis, the association between the EASIX score and the probability of NRP demonstrated a significant nonlinearity. As illustrated in Figure 2, the predicted risk of NRP increased progressively with rising EASIX values. Comparison of the spline model with a simple linear model using a likelihood ratio test confirmed significant non-linearity (*p* for non-linearity <0.001).

### 3.3. Association Between EASIX Score and In-Hospital Mortality

Table 3 presents results from univariable logistic regression analyses evaluating factors associated with in-hospital mortality. In univariable analyses, a broad range of demographic, clinical, procedural, and laboratory variables were significantly associated with in-hospital death, including advanced age, cardiac arrest on admission, DM, HF, CRF, hemodialysis, COPD, NRP, and several markers of myocardial injury, renal dysfunction, inflammation, and hematologic status. In addition, lower LVEF, reduced eGFR, higher blood glucose, admission troponin, LDH, C-reactive protein (CRP), WBC count, neutrophil count, and the EASIX score were significantly associated with increased in-hospital mortality. Procedural characteristics such as stent diameter were also significantly associated with mortality, whereas culprit lesion location, stent length, and symptom-to-balloon time were not.

Table 4 summarizes results from multiple multivariable logistic regression models assessing the independent association of the EASIX score with in-hospital mortality across clinical domains. Across all models, the EASIX score remained independently and consistently associated with in-hospital mortality, with adjusted ORs ranging from 1.371 to 2.096, all of which were statistically significant (*p* < 0.001). The association persisted after adjustment for key clinical factors, including age, NRP, LVEF, cardiac arrest on admission, DM, HF, renal function parameters, metabolic markers, inflammatory indices, myocardial injury markers, culprit vessel involvement, and procedural characteristics. Notably, the strength and direction of the association between EASIX and in-hospital mortality remained stable across all model configurations. In addition to the consistent adjusted associations observed in multivariable analyses, the incremental prognostic value of the EASIX score was further supported by reclassification analyses. Across the evaluated multivariable models, the inclusion of EASIX yielded consistently positive NRI and IDI values.

**Figure 3:**
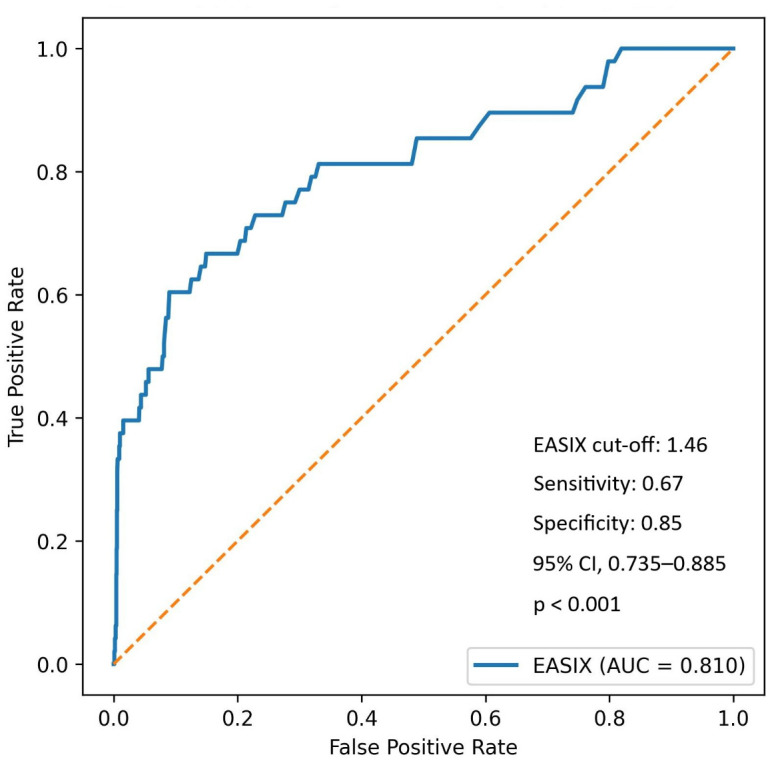
Receiver Operating Characteristic curve for the EASIX score predicting in-hospital mortality (AUC = 0.810). Abbreviations: AUC, area under the curve; CI, confidence interval; EASIX, endothelial activation and stress index. The orange dashed line represents the reference line corresponding to an AUC of 0.5, indicating random classification performance.

Using Youden’s index, an optimal EASIX cut-off of 1.46 yielded a sensitivity of 66.7% and a specificity of 85.1% for predicting in-hospital mortality. The AUC was 0.810 (95% CI, 0.735–0.885; *p* < 0.001), with positive and negative predictive values of 10.2% and 99.0%, respectively (Figure 3).

**Figure 4:**
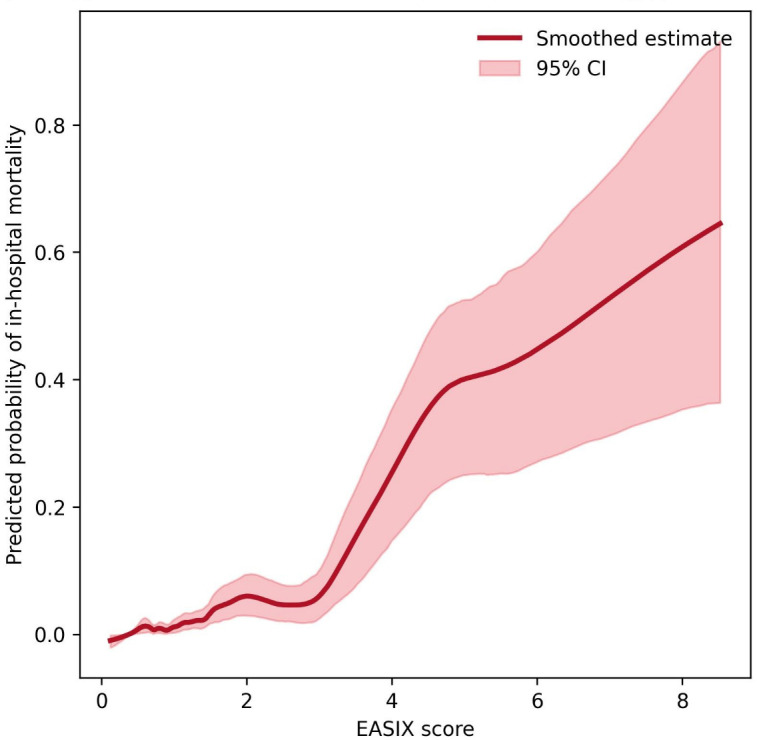
Smoothed non-linear association between EASIX score and in-hospital mortality. Abbreviations: CI, confidence interval; EASIX, endothelial activation and stress index.

A LOWESS analysis showed a nonlinear association between the EASIX score and in-hospital mortality, with progressively increasing risk at higher EASIX values (Figure 4). Given the limited number of mortality events and the strongly monotonic relationship between EASIX and in-hospital death, nonlinearity was explored using smoothed curve analysis. The analysis revealed a marked increase in the predicted probability of in-hospital mortality with higher EASIX scores, consistent with the multivariable regression models.

**Figure 5:**
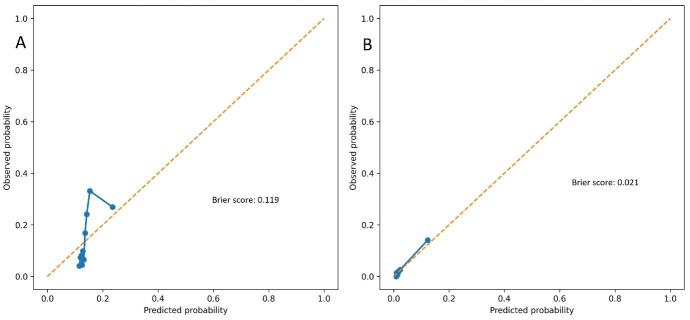
(**A**) Calibration plot for prediction of the no-reflow phenomenon; (**B**) Calibration plot for prediction of in-hospital mortality. Blue dots represent the observed event proportions within predicted probability bins, and the solid blue line connects these points to illustrate model calibration. The orange dashed line indicates the ideal calibration line where predicted probabilities equal observed probabilities.

Model calibration was evaluated using calibration plots and Brier scores. The EASIX-based model showed acceptable calibration for predicting the NRP, with a Brier score of 0.119 (Figure 5A). For in-hospital mortality, the model demonstrated excellent calibration, with a low Brier score of 0.021, indicating high overall predictive accuracy despite the limited number of events (Figure 5B).

### 3.4. Clinical Characteristics According to EASIX Tertiles

Table 5 presents the clinical, procedural, and laboratory characteristics of the study population, stratified by EASIX tertiles. Patients in higher EASIX tertiles were progressively older, with median age increasing in a stepwise manner across tertiles. The proportion of male patients also increased significantly with higher EASIX categories. The frequency of cardiac arrest at admission differed significantly across tertiles, with the highest rate observed in patients in the upper EASIX tertile. Although the prevalence of DM, previous PCI, and COPD did not differ significantly among groups, higher EASIX tertiles were associated with increased rates of hypertension, HF, and CRF. Regarding angiographic and procedural characteristics, culprit vessel distribution showed a borderline difference across EASIX tertiles, whereas stent length and stent diameter were comparable among groups. LVEF did not significantly differ across EASIX tertiles. Laboratory analyses showed a graded rise in WBC counts with higher EASIX categories. Importantly, the prevalence of the NRP increased markedly across tertiles, from 5.4% in the lowest tertile to 28.7% in the highest tertile (*p* < 0.001). Similarly, in-hospital mortality was significantly higher in the upper EASIX tertile than in the lower and intermediate tertiles (*p* < 0.001).

### 3.5. Sensitivity and Interaction Analyses

To assess the consistency of EASIX as a predictor of NRP across clinically relevant subgroups, stratified univariable logistic regression analyses were performed according to ischemic time and baseline renal function. EASIX remained significantly associated with NRP in both patients with short ischemic time (<120 min) (OR: 1.533, 95% CI: [1.293–1.817], *p* < 0.001) and those with prolonged ischemic time (≥120 min) (OR: 1.234, 95% CI: [1.071–1.421], *p* = 0.004). Similarly, among patients with preserved renal function (eGFR >60 mL/min/1.73 m^2^), EASIX was a significant predictor of NRP (OR: 1.318, 95% CI: [1.139–1.525], *p* < 0.001), whereas the association was weaker but statistically significant in those with reduced renal function (eGFR ≤60) (OR: 1.038, 95% CI: [1.012–1.085], *p* = 0.021).

To explore potential effect modification, an interaction analysis was conducted using a logistic regression model incorporating an “EASIX × ischemic time” interaction term. The interaction term yielded an OR of 0.805 (95% CI: [0.627–1.033], *p* = 0.089), suggesting a trend toward differential predictive value of EASIX based on ischemic time, although not reaching conventional statistical significance.

## 4. Discussion

In this large cohort of patients with STEMI undergoing pPCI, the present study demonstrates a consistent, independent association between the EASIX and adverse early reperfusion outcomes. The findings indicate that a higher EASIX score is closely associated with both NRP and in-hospital mortality, independent of established clinical, laboratory, and procedural risk factors. By capturing systemic endothelial stress through routinely available biomarkers, EASIX appears to reflect underlying microvascular vulnerability that predisposes patients to impaired myocardial reperfusion and early mortality after STEMI.

Over recent years, it has emerged as a global marker of endothelial cell dysfunction in diverse settings [14]. This background provides a strong rationale for our findings—NRP is fundamentally a consequence of severe microvascular and endothelial injury in reperfused myocardium [15]. Failure to reperfuse tissue despite opening the epicardial artery reflects an interplay of endothelial damage, capillary obstruction, and distal microthrombosis [15]. EASIX captures these same pathological processes in the systemic circulation. A higher EASIX implies elevated endothelial stress and microangiopathic burden, which plausibly predisposes patients to the microvascular collapse characteristic of NRP [16]. Each component of EASIX has biologically relevant connections to NRP pathophysiology. LDH, a marker of cellular injury, rises with extensive tissue hypoxia and cell death, including endothelial cell damage [17]. During prolonged ischemia, endothelial cells accumulate catabolites such as lactate, leading to cell swelling and microvascular obstruction [15]. An elevated LDH thus signals widespread cell necrosis and endothelial damage [18], conditions known to precipitate NRP [15]. Creatinine, beyond indicating renal function, reflects systemic vascular injury from chronic inflammation and oxidative stress [19]. Even modest renal impairment creates a pro-inflammatory, hypercoagulable milieu that injures the endothelium [20]. In STEMI, patients with higher baseline creatinine have been shown to experience NRP more often [20]. This corresponds with our finding that the creatinine component of EASIX was higher in NRP patients, reinforcing the link between pre-existing endothelial dysfunction (via renal insufficiency) and impaired microvascular reperfusion. Finally, platelet count is inversely associated with microthrombus formation. Thrombocytopenia in a high-EASIX patient may reflect increased platelet consumption from diffuse endothelial activation and clot formation [21]. Notably, NRP is driven in part by distal embolization of platelet aggregates and fibrin into the microcirculation [22]. A lower platelet count (yielding a higher EASIX) can thus serve as a surrogate for this pathological microthrombotic process. Taken together, a high EASIX reflects a dangerous triad of endothelial cell injury, microvascular thrombus burden, and end-organ stress, aligning closely with the established mechanisms of NRP.

Our results also align with prior studies of inflammatory and endothelial biomarkers in NRP. Systemic inflammation is a key contributor to microvascular dysfunction. For instance, elevated high-sensitivity CRP has been identified as an independent predictor of NRP in STEMI [23], and a higher neutrophil–lymphocyte ratio correlates with slow-flow/NRP [24], underscoring a strong inflammatory component. EASIX may indirectly reflect this pro-inflammatory state: inflammation exacerbates tissue injury (raising LDH) and promotes thrombogenesis (depleting platelets), thereby increasing the EASIX score. Likewise, DM—a condition of chronic endothelial inflammation—is well known to predispose patients to NRP. Hyperglycemia and insulin resistance drive oxidative stress and an imbalance in vasoactive mediators, impairing endothelial function and fostering a hypercoagulable state [25]. In our cohort, EASIX remained an independent predictor of NRP after adjustment for conventional risk factors (age, DM, HF, and eGFR). This suggests that EASIX captures an aggregate risk attributable to endothelial pathology that extends beyond any single clinical variable.

Our findings align with emerging research on direct endothelial biomarkers in STEMI. Endocan is a soluble proteoglycan released during endothelial activation. Recent studies have shown that higher serum endocan levels are independent predictors of angiographic NRP in pPCI-treated STEMI patients [26]. A review of seven clinical studies found that elevated endocan was strongly associated with impaired reperfusion, no-reflow occurrence (OR~2.4), and worse cardiac outcomes [26]. This parallels our observation that an endothelial stress index, such as EASIX, predicts NRP, reinforcing the central role of endothelial dysfunction in this complication. Notably, while endocan requires specialized assays, EASIX is calculated from routine lab tests and is therefore readily available at no extra cost or delay. EASIX may thus be a practical tool for risk stratification. By applying EASIX, clinicians could identify high- or low-risk patients for NRP early, prompting consideration of preventive measures in the catheterization laboratory.

In addition to predicting NRP, we found that higher admission EASIX scores were associated with significantly increased in-hospital mortality. This aligns with multiple studies reporting the prognostic value of EASIX in cardiovascular populations. Sang et al. observed that among intensive care unit patients with acute myocardial infarction, an elevated EASIX was linked to a ~70% higher risk of 30-day mortality [27]. Similarly, Finke et al. validated EASIX as an independent predictor of long-term mortality in patients with coronary artery disease undergoing catheterization (per log2 increase: HR~1.5) [28]. The consistent message is that EASIX stratifies patients by the overall severity of endotheliopathy, which translates into differential survival outcomes [29]. Our data extend this evidence to the acute phase of STEMI: patients with high EASIX are not only prone to NRP but also at heightened risk of early mortality. Indeed, NRP itself is a known harbinger of adverse events, including acute HF, cardiogenic shock, and life-threatening arrhythmias in the setting of myocardial infarction [30]. It follows that patients with both a high EASIX and NRP represent a particularly vulnerable subgroup. Even those with high EASIX who avoid NRP may have diffuse endothelial dysfunction that can manifest as other complications, contributing to worse short-term outcomes.

The acceptable prognostic performance of EASIX in our study underscores the close link between systemic endothelial health and cardiac outcomes after STEMI. EASIX, a composite index of indirect endothelial activation and microvascular stress, appears to capture many pathophysiological factors that standard risk markers address only in isolation. It not only correlates with known predictors of NRP but also provides additive predictive value by reflecting the combined effects of these derangements. From a clinical perspective, incorporating EASIX into STEMI risk assessment could enhance our ability to anticipate complications. Ultimately, our findings highlight that microvascular and endothelial factors are as critical as epicardial artery patency in determining patient outcomes. By validating EASIX as a predictor of NRP and early mortality, we add to the growing body of literature advocating the use of endothelial biomarkers in cardiovascular risk stratification. This novel insight invites further research to confirm EASIX’s utility in larger, prospective cohorts and to explore whether interventions that improve endothelial function can translate into lower NRP incidence and better survival.

### Study Limitations

This study has several limitations. First, its retrospective, single-center design may introduce selection bias and limit the generalizability of the findings. Second, because EASIX was calculated solely from admission laboratory data, temporal changes in endothelial stress and its dynamic interactions with microvascular dysfunction could not be assessed. Third, invasive or advanced imaging-based assessments of microcirculatory function—such as the index of microcirculatory resistance, coronary flow reserve, or cardiac magnetic resonance imaging—were unavailable, preventing direct confirmation of microvascular obstruction. Additionally, while our exclusion criteria aimed to minimize confounding from systemic inflammatory, infectious, hepatic, or hematologic conditions, the number of patients excluded on this basis was relatively small (*n* = 17), and thus unlikely to meaningfully impact generalizability. Although EASIX demonstrated good predictive performance for both NRP and in-hospital mortality, we were unable to directly compare it with existing NRP risk scores because several score-specific variables were missing (e.g., thrombus burden, Killip class, pre-PCI TIMI flow). Future prospective research with complete angiographic and hemodynamic datasets is warranted to evaluate how EASIX performs compared with, or in combination with, established risk stratification tools. In addition, although our findings support the prognostic value of EASIX, the present study was not designed to evaluate its clinical utility or to guide management decisions. Notably, this study focused exclusively on STEMI patients undergoing pPCI and did not include patients with non-ST-elevation myocardial infarction (NSTEMI). We deliberately limited the population to STEMI due to its more homogeneous presentation and standardized management pathways. In contrast, NSTEMI encompasses a more heterogeneous clinical spectrum, including patients managed conservatively, those undergoing delayed intervention, and those deemed unsuitable for PCI. As such, the timing and context of EASIX measurement—and its relationship with procedural outcomes like NRP—may be less consistent in NSTEMI, potentially confounding interpretation. Nonetheless, future studies are needed to explore whether EASIX retains similar prognostic relevance in NSTEMI cohorts. Lastly, although extensive multivariable adjustments were applied, residual confounding cannot be ruled out entirely. Despite these limitations, the large cohort size, consistent treatment protocols, and appropriate statistical analyses support the validity and potential applicability of our findings.

## 5. Conclusions

This study demonstrates, for the first time, that the EASIX is independently associated with both coronary NRP and in-hospital mortality in patients with STEMI undergoing pPCI. By reflecting systemic endothelial stress through accessible laboratory parameters, EASIX offers a pathophysiologically plausible and clinically practical tool for early risk stratification in STEMI. Our findings highlight the potential utility of EASIX in identifying high- or low-risk individuals at admission, before invasive management is initiated. Given its reproducibility, low cost, and integrative nature, EASIX may serve as a valuable adjunct to conventional risk assessment models. Nevertheless, external validation through prospective, multicenter studies is warranted to confirm these observations and to explore whether EASIX-guided strategies can improve clinical outcomes.

## Figures and Tables

**Table 1:** Baseline demographic, clinical, procedural, and laboratory characteristics of patients with and without the no-reflow phenomenon.

Variables	Patients Without NRP (*n* = 1659)	Patients with NRP (*n* = 272)	*p*-Value
Demographics			
Age, years	56 (49–65)	59 (51–68)	0.001
Male sex, *n* (%)	1377 (83.0)	223 (82.0)	0.680
Smoking, *n* (%)	734 (44.2)	111 (40.8)	0.290
Comorbidities, *n* (%)			
Cardiac arrest on admission	76 (4.6)	30 (11.0)	<0.001
Diabetes mellitus	379 (22.8)	72 (26.5)	0.190
Hypertension	503 (30.3)	87 (30.2)	0.580
Heart failure	62 (3.7)	24 (8.8)	<0.001
Chronic renal failure	22 (1.3)	10 (3.7)	0.005
Hemodialysis	11 (0.7)	9 (3.3)	<0.001
Previous PCI	211 (12.7)	37 (13.6)	0.686
COPD	15 (0.9)	3 (1.1)	0.752
Periprocedural characteristics			
Culprit lesion, *n* (%)			0.630
LMCA	1 (0.1)	0 (0)	
LAD	774 (46.7)	112 (41.2)	
LCx	222 (13.4)	45 (16.5)	
RCA	628 (37.9)	108 (39.7)	
Other vessels	34 (2.0)	7 (2.6)	
Stent length, mm	21.1 ± 6.3	21.8 ± 6.2	0.110
Stent diameter, mm	3.1 ± 0.8	3.1 ± 0.4	0.692
Patients receiving tirofiban, *n* (%)	862 (52.0)	138 (50.7)	0.708
Symptom to balloon time, min	180 (120–300)	210 (120–360)	0.002
TIMI myocardial perfusion grade (post-PCI)	3 (3–3)	1 (0–2)	<0.001
Baseline medications, *n* (%)			
β-blocker	389 (23.9)	67 (25.4)	0.608
ACEi or ARB	540 (33.2)	85 (31.3)	0.691
Statin	635 (40.1)	100 (38.9)	0.715
Laboratory findings			
LVEF, %	50 (40–55)	45 (35–55)	0.001
Serum creatinine, mg/dL	0.8 (0.7–1.0)	1.0 (0.8–1.2)	<0.001
eGFR, mL/min/1.73 m^2^	95.6 (81.1–105.2)	88.1 (67.6–98.9)	<0.001
Serum sodium, mmol/L	136.0 (134.0–139.0)	136.5 (134.0–139.0)	0.679
Serum albumin, g/dL	3.7 (3.4–3.9)	3.7 (3.4–3.9)	0.236
Blood glucose, mg/dL	132 (112–170)	138 (116–191)	0.020
Triglycerides, mg/dL	137 (97–189)	135 (101–190)	0.755
HDL-cholesterol, mg/dL	37 (31–43)	38 (31–43)	0.721
LDL-cholesterol, mg/dL	111 (87–137)	105 (87–139)	0.388
Admission troponin, ng/L	6.1 (0.9–27.7)	16.6 (10.6–33.2)	<0.001
Laktat dehidrogenase, U/L	224 (179–267)	256 (204–332)	<0.001
AST, U/L	63 (31–131)	64 (31–154)	0.566
ALT, U/L	31 (21–47)	31 (21–52)	0.361
C-reactive protein, mg/L	7 (4–8)	7 (5–9)	0.598
White blood cells, ×10^3^/mm^3^	11.7 (9.5–14.0)	12.1 (9.6–15.9)	0.010
Hemoglobin, g/dL	13.8 (12.6–14.9)	13.7 (12.7–14.8)	0.402
Platelets, ×10^3^/mm^3^	233 (199–278)	214 (176–251)	<0.001
Neutrophils, ×10^3^/mm^3^	9.0 (6.9–11.4)	8.9 (6.5–11.9)	0.649
Lymphocytes, ×10^3^/mm^3^	1.6 (1.2–2.3)	1.6 (1.2–2.3)	0.953
EASIX	0.80 (0.59–1.12)	1.21 (0.87–1.59)	<0.001
Length of hospitalization, days	3 (3–4)	3 (3–4)	0.785
In-hospital mortality, *n* (%)	25 (1.5)	23 (8.5)	<0.001

Abbreviations: ACEi, angiotensin-converting enzyme inhibitor; ALT, alanine aminotransferase; ARB, angiotensin II receptor blocker; AST, aspartate aminotransferase; COPD, chronic obstructive pulmonary disease; EASIX, endothelial activation and stress index; eGFR, estimated glomerular filtration rate; HDL, high-density lipoprotein; LAD, left anterior descending artery; LCx, left circumflex artery; LDL, low-density lipoprotein; LMCA, left main coronary artery; LVEF, left ventricular ejection fraction; NRP, no-reflow phenomenon; PCI, percutaneous coronary intervention; RCA, right coronary artery; TIMI, thrombolysis in myocardial infarction.

**Table 2:** Univariable and multivariable logistic regression analysis for the prediction of no-reflow phenomenon.

	Univariable Regression			Multivariable Regression		
Variable	Odds Ratio (OR)	95% Confidence Interval	*p*-Value	Odds Ratio (OR)	95% Confidence Interval	*p*-Value
Age (per year)	1.019	1.008–1.030	<0.001	1.008	0.993–1.024	0.280
Cardiac arrest on admission	2.581	1.662–4.021	<0.001	1.379	0.800–2.375	0.247
Heart failure ^§^	2.493	1.530–4.071	<0.001	-	-	-
Chronic renal failure ^‡^	2.840	1.331–6.072	0.007	-	-	-
Hemodialysis ^‡^	5.127	2.100–12.491	<0.001	-	-	-
Symptom-to-balloon time (per min)	1.002	1.001–1.003	<0.001	1.002	1.001–1.003	<0.001
LVEF (per %) ^§^	0.976	0.963–0.989	<0.001	0.994	0.980–1.009	0.449
Serum creatinine ^‡ †^	3.099	2.131–4.501	<0.001	-	-	-
eGFR (per mL/min/1.73 m^2^) ^‡^	0.982	0.976–0.987	<0.001	0.993	0.985–1.002	0.122
Blood glucose	1.002	1.001–1.004	0.001	1.000	0.999–1.002	0.726
Admission troponin	1.014	1.007–1.020	<0.001	1.012	1.004–1.019	0.002
LDH ^†^	1.002	1.001–1.002	<0.001	-	-	-
White blood cells	1.065	1.037–1.094	<0.001	1.069	1.033–1.106	<0.001
Platelets ^†^	0.995	0.993–0.998	<0.001	-	-	-
EASIX score ^†^	1.328	1.188–1.484	<0.001	1.485	1.286–1.715	<0.001

Abbreviations: EASIX, endothelial activation and stress index; eGFR, estimated glomerular filtration rate; LDH, lactate dehydrogenase; LVEF, left ventricular ejection fraction. Variables marked with ^§^ were not simultaneously included in the multivariable model due to strong clinical and pathophysiological overlap. LVEF was preferred over heart failure status to better reflect systolic function. Variables marked with ^‡^ represent closely related renal function parameters. To avoid redundancy and collinearity, eGFR was selected as a continuous integrative marker of renal function, whereas chronic renal failure and hemodialysis were excluded from the multivariable analysis. Variables marked with ^†^ denote components of the EASIX. As EASIX is a composite score derived from serum creatinine, LDH, and platelet count, these individual parameters were not entered simultaneously into the multivariable model to prevent multicollinearity. Multicollinearity was assessed using variance inflation factors (VIFs). All variables retained in the final multivariable model demonstrated low VIF values (<3.0), indicating the absence of clinically relevant multicollinearity.

**Table 3:** Univariable logistic regression analysis for the prediction of in-hospital mortality.

Variable	Odds Ratio	95% Confidence Interval	*p*-Value
Age (per year)	1.061	1.036–1.088	<0.001
Cardiac arrest on admission	21.963	11.963–40.323	<0.001
Diabetes mellitus	2.868	1.609–5.111	<0.001
Heart failure	5.413	2.532–11.572	<0.001
Chronic renal failure	15.492	6.560–36.583	<0.001
Hemodialysis	19.071	6.987–52.054	<0.001
COPD	8.302	2.321–29.693	0.001
No-reflow phenomenon	6.037	3.374–10.802	<0.001
Culprit lesion (LAD)	1.086	0.612–1.928	0.777
Stent length	0.956	0.901–1.013	0.126
Stent diameter	0.188	0.064–0.554	0.002
Symptom to balloon time	1.001	0.999–1.003	0.186
LVEF (per %)	0.911	0.883–0.939	<0.001
Serum creatinine	3.102	2.132–4.504	<0.001
eGFR (per mL/min/1.73 m^2^)	0.950	0.938–0.961	<0.001
Blood glucose (per mg/dL)	1.007	1.004–1.009	<0.001
Admission troponin	1.010	1.002–1.018	0.015
LDH	1.002	1.001–1.003	0.001
C-reactive protein	1.009	1.003–1.015	0.005
White blood cells	1.123	1.071–1.176	<0.001
Platelets	0.993	0.988–0.998	0.004
Neutrophils	1.110	1.052–1.172	<0.001
EASIX score	1.645	1.412–1.916	<0.001

Abbreviations: COPD, chronic obstructive pulmonary disease; EASIX, endothelial activation and stress index; eGFR, estimated glomerular filtration rate; LAD, left anterior descending artery; LDH, lactate dehydrogenase; LVEF, left ventricular ejection fraction.

**Table 4:** Independent association of EASIX score with in-hospital mortality in multiple multivariable models ^†^.

Model	Adjusted OR (EASIX)	95% Confidence Interval	*p*-Value	NRI (for EASIX)	IDI (for EASIX)
Model 1					
(Clinical: age + NRP + LVEF + EASIX)	1.804	1.480–2.200	<0.001	0.785	0.074
Model 2					
(Clinical: age + male sex + cardiac arrest + EASIX)	1.576	1.332–1.864	<0.001	0.836	0.066
Model 3					
(Clinical: age + diabetes mellitus + heart failure + EASIX)	1.618	1.381–1.895	<0.001	1.000	0.068
Model 4					
(Renal–metabolic: age + eGFR + blood glucose + EASIX)	1.371	1.159–1.623	<0.001	0.660	0.046
Model 5					
(Inflammatory: age + white blood cells + C-reactive protein + EASIX)	1.621	1.382–1.901	<0.001	0.942	0.070
Model 6					
(Myocardial injury/infarct anatomy: age + troponin + culprit LAD + EASIX)	1.558	1.338–1.814	<0.001	0.798	0.070
Model 7					
(Procedural: age + symptom-to-balloon time + stent diameter + EASIX)	2.096	1.728–2.541	<0.001	0.902	0.165
Model 8					
(Hemodynamic: age + cardiac arrest + NRP + EASIX)	1.513	1.263–1.812	<0.001	0.935	0.058

Abbreviations: EASIX, endothelial activation and stress index; eGFR, estimated glomerular filtration rate; LAD, left anterior descending artery; NRP, no-reflow phenomenon. ^†^ Multivariable logistic regression analyses were performed using clinically and biologically distinct model configurations to account for the limited number of in-hospital mortality events and to minimize the risk of model overfitting. Each model incorporated the EASIX score, along with up to 3 additional covariates selected from different clinical domains. Adjusted odds ratios (ORs) with 95% confidence intervals are reported for EASIX across all models.

**Table 5:** Clinical, procedural, and laboratory characteristics according to EASIX tertiles.

Variables	EASIX < 0.607 (*n* = 478)	EASIX 0.607–1.200 (*n* = 975)	EASIX > 1.200 (*n* = 478)	*p*-Value
Age, years	53 (46–63) ^a^	57 (50–65) ^b^	60 (51–70) ^c^	<0.001
Cardiac arrest on admission	24 (5.0) ^a^	41 (4.2) ^a^	41 (8.6) ^b^	0.002
Male sex, *n* (%)	365 (76.4) ^a^	807 (82.8) ^b^	428 (89.5) ^c^	<0.001
Diabetes mellitus, *n* (%)	100 (20.9)	230 (23.6)	121 (25.3)	0.268
Hypertension, *n* (%)	127 (26.6) ^a^	298 (30.6) ^a,b^	165 (34.5) ^b^	0.028
Heart failure, *n* (%)	11 (2.3) ^a^	47 (4.8) ^a,b^	28 (5.9) ^b^	0.021
Chronic renal failure, *n* (%)	3 (0.6) ^a^	8 (0.8) ^a^	21 (4.4) ^b^	<0.001
Previous PCI, *n* (%)	55 (11.5)	123 (12.6)	70 (14.6)	0.334
COPD, *n* (%)	3 (0.6)	8 (0.8)	7 (1.5)	0.354
Culprit lesion, *n* (%)				0.052
LMCA	1 (0.2)	0 (0)	0 (0)	
LAD	225 (47.1)	467 (47.9)	194 (40.6)	
LCx	71 (14.9)	128 (13.1)	68 (14.2)	
RCA	173 (36.2)	359 (36.8)	204 (42.7)	
Other vessels	8 (1.7)	21 (2.2)	12 (2.5)	
Stent length, mm	20.8 ± 6.2	21.0 ± 6.3	21.7 ± 6.3	0.846
Stent diameter, mm	3.11 ± 0.39	3.12 ± 1.02	3.10 ± 0.39	0.093
LVEF, %	50 (40–55)	50 (40–55)	48 (40–55)	0.167
White blood cells, *×*10^3^/mm^3^	11.0 (9.1–13.6) ^a^	11.7 (9.5–14.1) ^b^	12.3 (10.3–15.0) ^c^	<0.001
NRP, *n* (%)	26 (5.4) ^a^	109 (11.2) ^b^	137 (28.7) ^c^	<0.001
In-hospital mortality, *n* (%)	4 (0.8) ^a^	17 (1.7) ^a^	27 (5.6) ^b^	<0.001

Abbreviations: COPD, chronic obstructive pulmonary disease; EASIX, endothelial activation and stress index; LAD, left anterior descending artery; LCx, left circumflex artery; LMCA, left main coronary artery; LVEF, left ventricular ejection fraction; NRP, no-reflow phenomenon; PCI, percutaneous coronary intervention; RCA, right coronary artery. Different superscript letters indicate statistically significant differences between groups.

## Data Availability

Data are available from the corresponding author upon reasonable request. The data are not publicly available due to privacy or ethical restrictions.
